# Visual Functional–Structural Plant Modeling Innovatively as a Compound Eye: Opening a New Way for Advancing the Scientific Cognition of Plant Vision

**DOI:** 10.1002/advs.202303399

**Published:** 2023-10-24

**Authors:** Yi Lin

**Affiliations:** ^1^ School of Earth and Space Sciences Peking University No. 5 Yiheyuan Road Beijing 100871 China

**Keywords:** basic‐pattern‐oriented modeling, compound eyes, crown shyness, omnidirectional surfaces, Pareto front branching, plant vision, visual functional–structural plant modeling

## Abstract

Plant vision is an interesting interdisciplinary branch of botany and vision science, and its emerging studies have composed an epic journey of discovery. However, there are few endeavors on modeling how a plant as an integrity sees. Inspired by the similarity between those discovered laws of plant vision and the visual performance of some insect species with compound eyes, the visual functional–structural plant modeling as a compound eye is innovatively proposed. Using this adapted basic‐pattern‐oriented modeling, we tried to validate its feasibility in terms of the structural support, visual pathway, and functional performance. First, for a diversity of woody plants, their crowns proved to show self‐similar profiles, which render the omnidirectional surfaces for structurally supporting the proposed model. Second, for many plant species, their branching proved to abide by the Pareto front, which ensures the optimality of assuming the visual pathway along the branching network. Third, in canopies the varying, but existing horizontal and vertical modes of crown shyness are detected, which in functional performance accords with the panoramic visibility of the proposed model. Overall, the feasibility of compound eye modeling is validated preliminarily, with the implication of opening a way for advancing the scientific cognition of plant vision.

## Graphical abstract

Plant vision is an emerging interdisciplinary branch of botany and vision science, but there are few endeavors on modeling how a plant as an integrity sees. The visual functional–structural plant modeling as a compound eye is innovatively proposed, with the implication of opening a new way for advancing the scientific cognition of plant vision.

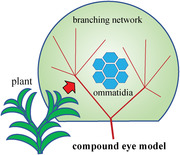

## 1 Introduction

Plant vision is a novel interdisciplinary branch of botany and vision science, and its research can tackle some relevant confusing secrets about life nature.^[1^ ^]^ With the potential significance in revolutionizing our scientific cognition of plants, its emerging research has composed an epic journey of discovery.^[^ ^1^, ^2^, ^3^, ^4^, ^5^ ^]^ Earlier, scientists observed that plants tend to grow toward the direction of sunlight and termed this effect as phototropism or “escape from shade”.^[1^ ^]^ Next, the chemical molecules, such as phytochrome, cryptochrome, and phototropin proved to act as the photoreceptors of plants.^[2^ ^]^ Then, Gianoli and Carrasco‐Urra reported that the leaves of a vine species can mimic those of different plant species in Chile's rainforests.^[3^ ^]^ After expelling those suppositions of its mimic mechanisms as the perception of either emission of volatile substances or microorganism‐based transferring of genes, Baluška and Mancuso speculated that the mimic function might come true based on the ocelli‐based visual capacity.^[4^ ^]^ Recently, this speculation has been basically verified experimentally.^[5^ ^]^ As mirrored by this admirable course of achieving novel findings, more knowledge about plant vision has kept being pursued.

However, how a plant as an integrity operates harmoniously with all sorts of its visual functions has not been figured out so far. In retrospect, the Austrian botanist Gottlieb Haberlandt once imaginatively hypothesized that plants may use their epidermal cells, just as we use our cornea and lens, to reconstruct the images of their growing external environments; however, he could not experimentally test this hypothesis.^[1^ ^]^ For the biochemical or genetic determinants, molecular biology is an important kind of biotechnology for exploring them, but it cannot answer the goal question. For example, people have not yet explained how the different types of photoreceptors, such as phytochromes, cryptochromes, and phototropins^[^ ^2^ ^]^ play their roles coordinatively. As to the modes of plants coordinating their visual functions, how the leaves of the vine species can mimic those of different plant species growing almost irregularly in the wild^[^ ^3^ ^]^ has been a puzzle. These cases suggested that the functional mechanism of a plant as an integrity running its vision systematically has been unclear yet.

Aiming at this discipline gap, we first made a theoretically abstract comparison of the major life species, namely, plants, low‐grade insects, high‐grade animals, and human beings, in terms of vision. Given high‐grade animals and human beings possess perspective eyes, it is a logically inevitable choice to pursue the visual similarity between low‐grade insects and plants. As we know, several low‐grade insect species have compound eyes.^[^ ^6^, ^7^, ^8^, ^9^, ^10^ ^]^ Like a fencer's mask in form, a compound eye is composed of many functional units repeatedly, each called ommatidia—an independent light receptor.^[1^ ^]^ Compound eyes present the extremely‐large fields of view, low aberrations and distortions, high temporal resolutions, and infinite depths of field.^[1^ ^]^ This functional mode of visuality can be detected in plants. Even the youngest parts of plant branches, tendrils, wood, shoots, and shoot tips are rich in photoreceptors as if a plant were covered by a crowd of tiny eyes.^[1^ ^]^ This strategy of avoiding the concentration of the visual function in a single area of a plant enlightened us to refer to compound eyes for characterizing its vision.

## 2 Results and Discussion

### 2.1 Visual Functional–Structural Plant Modeling as a Compound Eye

Our comparison of plants and compound eyes in vision showed their functional similarity. Plants have proved to be able to sense the ultraviolet, blue, green, red, and infrared spectra,^[11^ ^]^ perceive collect the information about the directions from which light rays come, as well as about their qualities,^[12^ ^]^ and measure the polarization traits;^[^ ^13^ ^]^ while for many insect species, their compound eyes have proved to be similarly able to sense the ultraviolet and blue‐red spectra,^[6^ ^]^ the information about the directions and qualities of light rays,^[7^ ^]^ and the polarization traits.^[8^ ^]^ As to the visual pathway that transmits the visual sensation from the perception end to the visual processing unit, the branching structure of plants is similar to the organization mode of the neural links in the compound eyes of some insect species.^[^ ^9^, ^10^ ^]^ The functional similarities in these two primary aspects of realizing vision suggested that we could assume a compound eye as the model for functionally characterizing how a plant as an integrity sees systematically.

**Figure 1:**
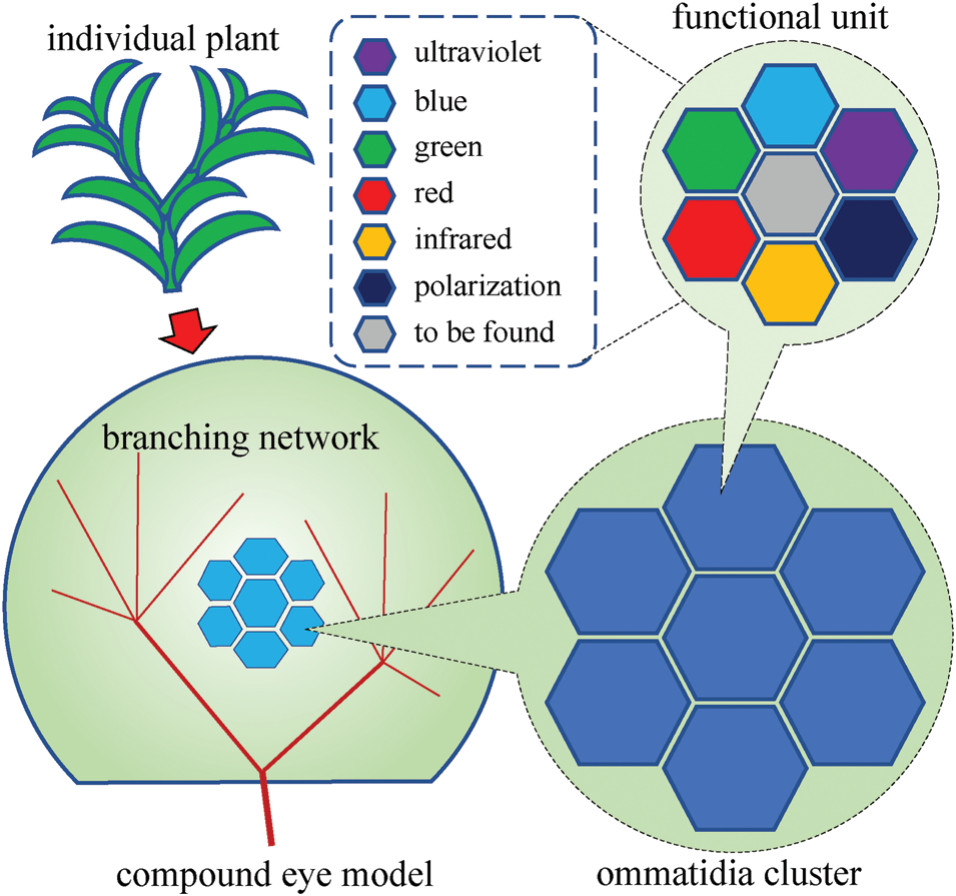
The schematic diagram of visual functional–structural plant modeling as a compound eye, in the pattern of combining the ommatidia clusters that each comprises the functional units (with the capacity of capturing the optical traits, such as ultraviolet, blue, green, red, infrared, and polarization and with the potential of embracing the optical traits to be found) for light sensing, with the branching network that enables transmitting the visual information for light signaling, which can functionally support plant visual perception and response, respectively.

The designed compound eye visual functional–structural plant model is schematically shown in **Figure** **1**. Such a theoretical model can functionally support the hypothesis about the visual foundation of the mimic function^[^ ^4^ ^]^ and its explanatory mechanism.^[5^ ^]^ Specifically, the vision of a plant is represented as a compound eye for modeling its visual sensing end, accompanied by a hierarchical branching network for modeling its visual response. The compound eye model is composed of ommatidium clusters at different scales, relating to the varying‐sized aggregations of stems, branches, and leaves. Further, each ommatidium can possess multiple functional units, which can individually sense the ultraviolet, blue, green, red, infrared, and polarization traits,^[^ ^11^, ^13^ ^]^ as illustrated in Figure 1. The mode of compound eye sensing can also be hierarchical, i.e., ranging from two tiny neighboring parts of a leaf with different inclination angles to the whole plant with a panoramic view. On the other hand, the hierarchical branching network proposed for characterizing plant's visual information transmitting ranges from the interlinks between such two neighboring tiny parts to the primary branches. The hierarchal branching network has its theoretical reasonability for characterizing how a plant transmits and processes its vision signals and shows its responding behaviors. This network model naturally following the mode of plant branching architecture can support both locally and globally transmitting the visual signals and running the principles of swarm intelligence, e.g., collective decision‐making,^[14^ ^]^ for making the optimal visual response.

### 2.2 Model Validation by Introducing Basic‐Pattern‐Oriented Modeling

**Figure 2:**
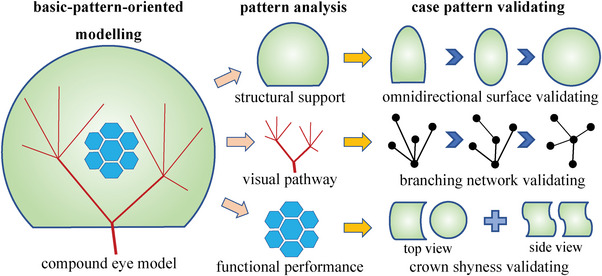
The schematic diagram of implementing our adapted method of basic‐pattern‐oriented modeling for testing our proposed compound eye model in terms of the basic patterns regarding structural support, visual pathway, and functional performance, specifically in the cases of omnidirectional surface validating, branching network validating, and crown shyness validating, respectively.

In principle, the initial comparison of plants and compound eyes in vision could serve as a preliminary theoretical validation of the proposed compound eye model, but this is not enough. For verifying and validating a proposed model, pattern‐oriented modeling is a typical state‐of‐the‐art kind of solution plan.^[15^ ^]^ Under this method framework, multiple verification patterns are used as filters for rejecting unrealistic model structures and parameter combinations, while a second, independent set of patterns is used for validation.^[15^ ^]^ In order to adapt this method to the validation of any model proposal at its initial stage, we converted it into a new version of basic‐pattern‐oriented modeling, i.e., with the basic patterns of the potential on validating the most original model examined. In this study, the basic patterns were specified to be structural support, visual pathway, and functional performance, as illustrated in **Figure** **2**. In terms of these basic patterns, the concrete validations of the proposed compound eye model were devised and performed as follows.

#### 2.2.1 Validation in Terms of Structural Support

**Figure 3:**
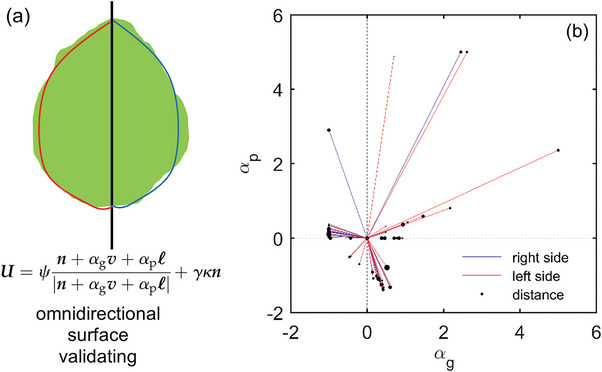
a) The schematic diagram of validating the proposed compound eye model in terms of structural support, in the case of whether the woody plant crowns present the omnidirectional surfaces as characterized by the propagating front model of tree growth^[^ ^12^ ^]^ (indicated by the listed formula, where *U* refers to the velocity of the front for drawing the crown envelope, *ψ* means the angle between the local tangent and the horizontal, *n* represents the outward normal unit vector, *ℓ* is the unit vector that represents the average direction of light, *v* is the upward vertical unit vector, *ĸ* is the in‐plane curvature, *γ* is a surface tension‐kind parameter acting on growth velocity, and *α*_g_ and *α*_p_ are the intensities of the crown gravitropic and phototropic responses, respectively), and b) the derived statistical distributions of the feature parameters (*α*_g_ and *α*_p_) characterizing the omnidirectional surfaces of crowns for a diversity of woody plant species from the results published by Duchemin et al.^[12^ ^]^

The first aspect of structural support was examined by case testing whether the crowns of the varying woody plant species present the omnidirectional surfaces, as featured by compound eyes. The testing was specifically operated by reanalyzing the derived profile characterizations of tree crown shapes that were based on the propagating front model of tree growth^[^ ^12^ ^]^ as shown in **Figure** **3**a. The formula delineating the velocity of the front was listed in Figure 3a, where *α*_g_ and *α*_p_ are the intensities of the crown gravitropic and phototropic responses, respectively. The propagating front is described by the homothetic surface,^[12^ ^]^ which models the crown envelope. Under this modeling framework, the testing was deployed to check if the omnidirectional surfaces can be derived from the propagating front models of crown envelopes for the varying *α*_g_‐*α*_p_ combination scenarios.

The result after statistics suggested that the omnidirectional surfaces can be detected from the crowns of the different woody plant species, as evidenced by the observation that the values of the model parameters (*α*_g_ and *α*_p_) are distributed in all four quadrants (Figure 3b). This discovery can lay the cornerstone for ensuring the structural support premise of the proposed compound eye model.

#### 2.2.2 Validation in Terms of Visual Pathway

**Figure 4:**
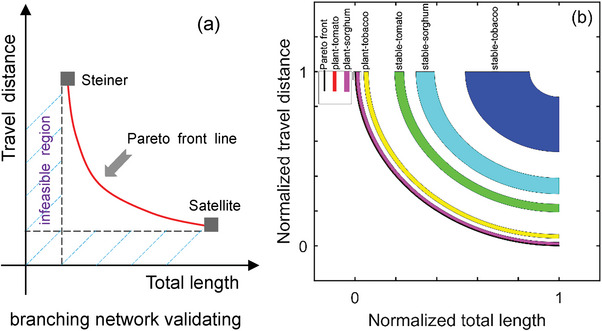
a) The schematic diagram of validating the proposed compound eye model in terms of the visual pathway, in the case of whether the branch network supporting it can abide by the Pareto front line^[^ ^16^ ^]^ when considering its two possible influence factors (travel distance and the total length, either of which takes the functional priority generates the Steiner and Satellite branching mode, respectively), and b) the derived statistical distributions of these two influence factors (normalized travel distance and normalized total length) characterizing the Pareto front‐followed modes of branching architectures for multiple plant species from the results published by Conn et al.^[16^ ^]^

The second aspect of the visual pathway was analyzed by testing whether the branching of plants is optimal for transmitting the visual information and making the visual response. In terms of the distance to the Pareto front line,^[16^ ^]^ we conducted the analysis of the optimality based on the considered factors of deciding plant architectures and the published results.^[16^ ^]^ Plant architectures are Pareto front optimal in a network cost versus performance trade‐off (**Figure** **4**a). This theory is often used in economics and engineering to find the satisfiable solutions that best trade off multiple, competing objectives.^[17^ ^]^ Recent advances in Pareto front analysis may be useful in this case to infer additional candidate objectives given architecture traits,^[18^ ^]^ and so do the branching network serving as the visual pathway.

The result indicated that the worst case of branching architecture in real plants is closer to the Pareto front than the best case of branching architecture represented by the stable model (in terms of the average distance ± standard error to the Pareto front: 5.45% ± 1.18 vs. 21.70% ± 2.31)^[^ ^16^ ^]^ (Figure 4b). Compared to the stable model,^[16^ ^]^ real plant branching architectures lie along the Pareto front for minimizing total branch length and nutrient transport distance—conferring a selective fitness advantage for the transport processes in plants,^[16^ ^]^ valid for visual information transmitting and processing as well. This case testification explicitly in terms of plant visual behaviors can set the foundation for guaranteeing the visual pathway premise of the proposed compound eye model.

#### 2.2.3 Validation in Terms of Functional Performance

After the functional premises proved to be guaranteed, the proposed theoretical model can be validated further from the perspective of its holistic effect characterized by plant behaviors. This effect can be exposed in the case of crown shyness.^[^ ^19^, ^20^ ^]^ After all, if the sensing foundation of crown shyness is the vision of plants, the situations of crown shyness can conversely mirror the functional validity of the proposed theoretical model. The theoretical rationale is to test if the models of compound eye and hierarchical branching network can explain the complicated relative spatial relationships between the crowns of any two neighboring plants.

**Figure 5:**
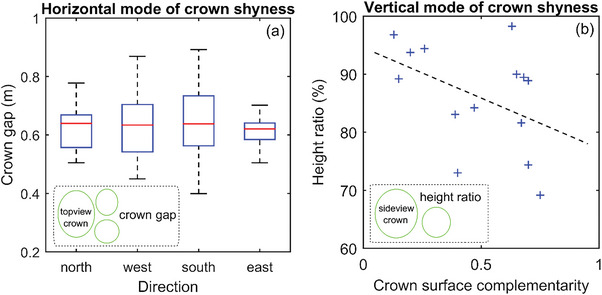
The crown shyness‐indicated results for validating our proposed compound eye model from the perspective of functional performance, specifically in the cases a) with the varying, but existing horizontal mode of crown shyness in terms of the top‐view crown gaps (range and mean, respectively) in different directions derived from the results published by Markham and Otárola^[^ ^19^ ^]^ and b) with the varying, but existing vertical mode of crown shyness in terms of the side‐view crown‐top‐indicated height ratio along with crown surface complementarity (diversity of height ratios and diversity of crown surface complementarities, respectively) derived from the results published by van der Zee et al.^[20^ ^]^ These newly derived results can be explained by the proposed compound eye model and, in turn, can verify its validity.

We disclosed the varying, but existing horizontal mode of crown shyness in terms of the top‐view crown gaps (range and mean, respectively) in the different directions (**Figure** **5**a), based on the data that was derived from the published wind‐crown gap ecological results.^[19^ ^]^ We unveiled the varying, but existing vertical mode of crown shyness in terms of the side‐view crown‐top‐indicated height ratio along with crown surface complementarity (diversity of height ratios and diversity of crown surface complementarities, respectively) (Figure 5b), based on the dataset that was derived from the published laser scanning results.^[20^ ^]^ These two decoded modes can be explained in principle by the proposed compound eye model and, in turn, can manifest its validity.

### 2.3 Functional Extensibility

**Figure 6:**
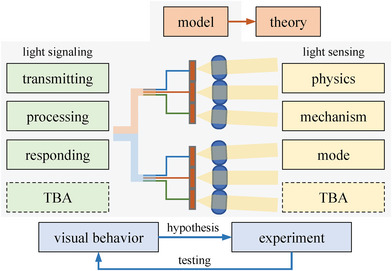
The schematic diagram of our proposed paradigm for guiding future research on the compound eye‐based visual functional–structural plant modeling, specifically including from model compositions (light sensing involves its physics, mechanism, mode, and TBA, while light signaling involves its processes of transmitting, processing, responding, and TBA), exploration approaches (hypothesis‐testing of plant visual behavior modeling based on experiments), to goal upgrading (from model characterization to theory derivation).

The proposed compound eye model was right from the outset endowed with functional extensibility, as illustrated in **Figure** **6**. The extensibility was first set at the functional aspect of light sensing, from bottom to top specifically involving the physics of ommatidia eye‐seeing, the mechanism of the compound eye‐seeing, and the mode of individual plant seeing. By extension, in the future, the possible discoveries at these levels or even at some distinctive aspects of the varying plant species growing in the diverse environments, as tabbed by the to‐be‐added (TBA) functional units (Figure 6), can be absorbed. The extensibility is also designed at the functional aspect of light signaling, from beginning to end specifically involving light signal transmitting, and processing. As marked by the TBA functional units (Figure 6), the proposed model was defined to incorporate the relevant discoveries about light signaling in plants. For example, the visual characteristics of algal ocelloids as the evolving origins of plant ocelli can also be considered for improving the model,^[21^ ^]^ and the visual pathway‐related branching network can be devised to be able to absorb the continuously upgraded swarm intelligence algorithms.^[22^ ^]^ Such functional extensibilities counted together can enhance the performance of the proposed compound eye model. With more improvements made, the model can further implement the more complicated functions, such as sensing light attenuation, enabling negative phototropism, and locating host plants.^[23^ ^]^ Thus, the theoretical model can accommodate future discoveries involving this topic and, the other way around, can also promote the relevant explorations in a guiding sense.

With the enhanced functions of light‐sensing and ‐signaling fused in the future, the proposed compound eye model, no doubt, can better characterize the diverse visual behaviors of plants. As shown in Figure 6, under the paradigm of hypothesis and testing, the coordination strategies assumed by different plants as individuals for achieving visions can be explored, and this top‐level visual modeling framework can be further optimized to pursue its best performance. This scheme can satisfy the core purpose of answering the goal question of how a plant as an integrity sees. Then, the optimal models may help stimulate enormous discussions and new experimental approaches for validating the similar‐theme studies in molecular and functional biology. This model has fundamental implications for inspiring more future studies from genetic dissection by biologists to computational biology by mathematicians, statisticians, and computer scientists with the purposes of deciphering more secrets about plant vision, e.g., setting up the perceptual foundation for stepping toward the 3D basic theories of plant forms.^[24^ ^]^ In the future, with the functional units exemplified in Figure 6 enhanced, the proposed theoretical model can serve as a cornerstone for supporting future explorations in this direction.

## 3 Conclusion

Our innovative proposal of compound eye modeling after validation can fill the basic gap of visual functional–structural plant modeling for characterizing how a plant as integrity sees. As a revolutionizing way for advancing the scientific cognition of plant vision, this model can help people to explore the novel laws and mechanisms of plant vision and then develop its new principles and theories, ranging from the specific ones available for the varying plant species to the general ones for all plant species. Further, it can promote extensively unveiling the novel secrets of life nature, e.g., the proactive‐perspective modes of the plant–plant, –environment, and –animal interactions.

## Conflict of Interest

The authors declare no conflict of interest.

## Data Availability

Data sharing is not applicable to this article as no new data were created or analyzed in this study.
